# Digital Self-Management Interventions for People With Osteoarthritis: Systematic Review With Meta-Analysis

**DOI:** 10.2196/15365

**Published:** 2020-07-20

**Authors:** Reza Safari, Jessica Jackson, David Sheffield

**Affiliations:** 1 Health and Social Care Research Centre University of Derby Derby United Kingdom; 2 University of Derby Online Learning Derby United Kingdom

**Keywords:** osteoarthritis, self-management, internet-based intervention, mobile phone, eHealth, mHealth, systematic review, meta-analysis

## Abstract

**Background:**

Osteoarthritis (OA) is not curable, but the symptoms can be managed through self-management programs (SMPs). Owing to the growing burden of OA on the health system and the need to ensure high-quality integrated services, delivering SMPs through digital technologies could be an economic and effective community-based approach.

**Objective:**

This study aims to analyze the effectiveness of digital-based structured SMPs on patient outcomes in people with OA.

**Methods:**

A total of 7 web-based and 3 gray literature databases were searched for randomized controlled trials assessing digital-based structured SMPs on self-reported outcomes including pain, physical function, disability, and health-related quality of life (QoL) in people with OA. Two reviewers independently screened the search results and reference lists of the identified papers and related reviews. Data on the intervention components and delivery and behavioral change techniques used were extracted. A meta-analysis, risk of bias sensitivity analysis, and subgroup analysis were performed where appropriate. The Grading of Recommendations, Assessment, Development, and Evaluations (GRADE) approach was used to assess the quality of evidence.

**Results:**

A total of 8 studies were included in this review involving 2687 patients with knee (n=2); knee, hip, or both (n=5); and unspecified joint (n=1) OA. SMPs were delivered via telephone plus audio and video, internet, or mobile apps. Studies reported that digital-based structured SMPs compared with the treatment as usual control group (n=7) resulted in a significant, homogeneous, medium reduction in pain and improvement in physical function (standardized mean difference [SMD] –0.28, 95% CI –0.38 to –0.18 and SMD –0.26, 95% CI –0.35 to –0.16, respectively) at posttreatment. The digital-based structured SMP effect on pain and function reduced slightly at the 12-month follow-up but remained to be medium and significant. The posttreatment effect of digital-based structured SMPs was small and significant for disability, but nonsignificant for QoL (SMD –0.10, 95% CI –0.17 to 0.03 and SMD –0.17, 95% CI –0.47 to 0.14, respectively; each reported in 1 study only). The 12-month follow-up effect of the intervention was very small for disability and QoL. The quality of evidence was rated as *moderate* for pain and physical function and *low* and *very low* for disability and QoL, respectively, using the GRADE approach.

**Conclusions:**

Digital-based structured SMPs may result in improvement in pain and physical function that is largely sustained at the 12-month follow-up in people with knee and hip OA. The effects on disability and QoL are smaller and less clear. The quality of evidence is moderate to low, and further research is required to confirm the findings of the review and assess the effects of digital-based structured SMPs on other health-related outcomes.

## Introduction

Osteoarthritis (OA) is a major burden for individuals, the health system, and the economy. The common symptoms of OA (ie, pain and subsequent physical inactivity) are risk factors in other chronic conditions, including cardiovascular problems, depression, stroke, cancers, and, consequently, premature mortality [1-6]. People with OA also experience fatigue, emotional distress, poor sleep, decreased productivity, social isolation, and poor quality of life (QoL) [7-9]. OA is not curable, but the pace of progression and symptoms can be managed. In a systematic review of guidelines for the management of OA [10], 12 of the 15 included guidelines made a strong recommendation for self-management and education. Evidence-based practice guidelines suggest that patients should be provided with tailored information to enhance understanding of the conditions and their progressive nature, tailored self-management programs (SMPs), information sharing, and regular contact with a multidisciplinary team to promote self-care and joint protection strategies [10-12].

SMPs are the structured and coordinated delivery of education and health behavior change interventions to empower people with OA to take care of their own condition [13,14]. Previous meta-analytic reviews show that SMPs and/or exercise interventions have small to medium benefits on health outcomes such as pain, function, and aspects of QoL [15-18]. A recent Cochrane review [19] revealed that, compared with usual care control groups, SMPs resulted in a significant but clinically unimportant reduction in pain up to 1 month postintervention (standardized mean difference [SMD] –0.26; 95% CI –0.41 to –0.10) and at 3- and 12-month follow-ups (SMD –0.17; 95% CI –0.26 to –0.08) [19]. No significant difference in patient-reported function was reported up to 1-month postintervention (SMD –0.01; 95% CI –019 to 018), but, at the 3- and 12-month follow-ups, function improved significantly in the SMP group (SMD –0.16; 95% CI –0.25 to –0.01). No effect of SMPs on QoL was reported in the review [19].

Due to the growing burden of arthritis on the health system, the increasing need to ensure high-quality integrated services; and the rapid advances in communication technology, health information, and services, delivering SMPs through digital technologies (eg, telephone, internet, mobile apps, and virtual reality equipment) could be an economical and effective community-based model of care [13,14]. Many people with OA continue to be in the workforce and have limited time to attend treatment sessions. In addition, people may live at a distance from physical health services [20,21]. Digital-based structured programs have the potential to be an important component in models of care for enhancing delivery to support self-management, offering a sustainable opportunity to improve patient outcomes, monitoring patients’ symptoms for intervention adaptation, and increasing access to best practice [21]. It could also address the lack of continuity of care, lack of self-management support, and difficulty in accessing allied health professionals and pain management specialists [22]. A World Health Organization (WHO) guideline recommends the use of digital interventions in an interlinked manner, among others, to improve individuals’ access to health services and information, and health workers can provide appropriate and high-quality care and can follow-up to ensure that individuals receive appropriate services [23].

There is a growing number of trials relating to digital-based structured interventions in OA conditions. A recent systematic review indicated that electronic health (eHealth; ie, internet, mobile, and telephone) exercise interventions, compared with no or other interventions, resulted in small effects in pain reduction, improved physical function, and improved health-related QoL [24]. Furthermore, moderate quality evidence indicates that telephone-based interventions (with educational material) reduce pain intensity and disability in people with OA of the knee or hip and spinal pain (back or neck pain) [25]. However, no review has focused explicitly on the effect of digital-based structured SMPs in people with OA. Therefore, this review aimed to determine the effectiveness of digital-based structured SMPs with controlled comparators on patient outcomes (pain, physical function, disability, and health-related QoL) in people with OA.

## Methods

### Systematic Literature Review

Guidance published in Preferred Reporting Items for Systematic reviews and Meta-Analyses [26] and the Cochrane Handbook of Systematic Reviews [27] was adhered to. The a priori protocol for the review is published in the International Prospective Register of Systematic Reviews (PROSPERO): CRD42018089322.

### Search Strategy

Studies were identified by searching web-based databases with support and consultation provided by an institutional librarian. The search included the Medical Literature Analysis and Retrieval System Online (MEDLINE), Excerpta Medica Database (EMBASE), Cochrane Central Register of Controlled Trials (CENTRAL), Cumulative Index to Nursing and Allied Health Literature (CINAHL), PsycINFO, Physiotherapy Evidence Database (PEDro), and PubMed and gray literature databases (Dissertation Abstracts International WorldCat, The Grey Literature Report, and Open Grey) using both Medical Subject Headings and free-text keywords relating to OA, digital-based structured self-management interventions, and outcomes stated below from inception to May 2018. Search strategies for the first 3 databases above and dates on which searches were conducted are listed in Multimedia Appendix 1. Searches of the reference list of the previous review papers and included studies were also conducted.

### Eligibility Criteria

#### Type of Studies

Randomized controlled trials (RCTs) of any design, including parallel-group, crossover, and cluster RCTs published in the English language were included in this review.

#### Participants

Adults (≥18 years of age) with a confirmed diagnosis of OA, either radiologically or by a health practitioner. All types of OA at any stage of the disease were considered. We included studies recruiting patients with OA with other conditions only if outcome data for OA patients were provided.

#### Intervention

The intervention considered structured and coordinated SMPs as defined by Lorig [14] and Osborne [13] in isolation or in combination with other interventions delivered fully or partially via digital technologies (eg, websites, mobile apps, social networking tools, web-based games, animation, and telephone). Self-management is defined as an engagement in activities that promote health and prevent adverse events; interacting with a health care professional; improving self-monitoring; coping with disease; and developing skills in problem-solving, decision making, resource utilization, forming of a patient and health care provider partnership, and taking action.

#### Control Condition

Any type of control group (ie, waitlist, treatment as usual or minimal interventions, alternative treatment, or other digital-based interventions) was considered.

#### Outcome

We included any psychometrically sound unidimensional or multidimensional measure as well as the relevant subscales relating to the following outcomes:

##### Primary Outcomes

1. Pain: Visual Analog Scale, Numerical Rating Scale, and Brief Pain Inventory.
2. Function: patient-specific physical function, physical function subscale of Arthritis Impact Measurement Scale, or Short Form-36.
3. Disability: Oswestry Disability Questionnaire and Roland-Morris Disability Questionnaire.

##### Secondary Outcomes

1. QoL: European Quality of Life-5 Dimensions, Short Form-36, or WHO Quality of Life–Brief scale.
2. Cost and resource use: We extracted the results of economics reported alongside the effectiveness studies, either full or partial economic evaluation or estimates of resource use and costs associated with interventions and comparators. Change from baseline data relating to any follow-up time points were considered.

### Study Selection and Data Extraction

Two reviewers (RS and JJ) independently screened abstracts and full text of the search results. Any disagreements were resolved through discussion. Data were extracted by 2 researchers (JJ and EH; mentioned in Acknowledgments) and cross-checked by a third researcher (RS) using a data extraction tool developed a priori based on the Cochrane Handbook recommendations. Data items included participants’ characteristics, guiding theory or rationale, mechanism of effect, the digital medium, method of delivery, who delivered, where it was delivered, adherence, and fidelity. Additional data relating to describing components of the interventions were extracted based on the Template for Intervention Description and Replication (TiDieR) guidance [28] and SMP components [13,14]. The behavior change techniques used were also extracted based on the hierarchical taxonomy by Michie et al [29] to examine if certain techniques were favored or, if there were sufficient data, whether particular techniques yielded bigger effects. The data extraction form was piloted on 2 included papers before full data extraction.

### Meta-Analysis

Outcome data were expressed as SMD and were pooled in a pair-wise fixed effects model stratified based on the outcome and type of digital medium. We used the mean (SD) of within-group change from baseline to calculate the SMDs. Per Cohen [30], SMDs <0.2 were classified as small, those between 0.2 and 0.8 were classified as medium, and those >0.8 were classified as large. We converted the SMD to the percentage change in the outcome measure by multiplying the SMD with the SD of the control group of the sufficiently powered study (ie, the trial with the largest sample size).

A sensitivity analysis was conducted for the risk of bias assessment. A subgroup analysis was performed to assess the effect of SMPs on the different digital media used, control conditions, and presence of active exercise component in the intervention. Heterogeneity across pooled studies was examined using the chi-squared test and I^2^ statistics. As there were <10 studies, we did not use a funnel plot or Egger test to assess publication bias. Furthermore, a narrative synthesis of the studies was conducted where there were insufficient data to pool studies in a meta-analysis. We did not conduct additional exploratory analyses to explore potential moderators because of an insufficient number of studies. The common study characteristics tables were supplemented by additional summary tables including the TiDieR process information; risk of bias assessment; effect estimates; Grading of Recommendations, Assessment, Development, and Evaluations (GRADE) analysis; and behavior change taxonomy groupings. We used Review Manager (RevMan, version 5.3; Cochrane Collaboration) for all analyses.

### Dealing With Missing Data

We contacted the authors of included studies for missing data. Aggregated data were provided by authors for 6 studies. We used the RevMan calculator to impute missing SDs from the test statistics reported for 2 studies.

### Risk of Bias and Grading of Recommendations, Assessment, Development, and Evaluations Assessment

Risk of bias was assessed using the risk of bias tool of the Cochrane Handbook for Systematic Reviews [27]. Quality of evidence for outcomes was assessed according to the 5 GRADE domains, including study limitation (risk of bias), inconsistency, indirectness, imprecision, and publication bias [31,32].

## Results

### Study Selection and Characteristics

**Figure 1:**
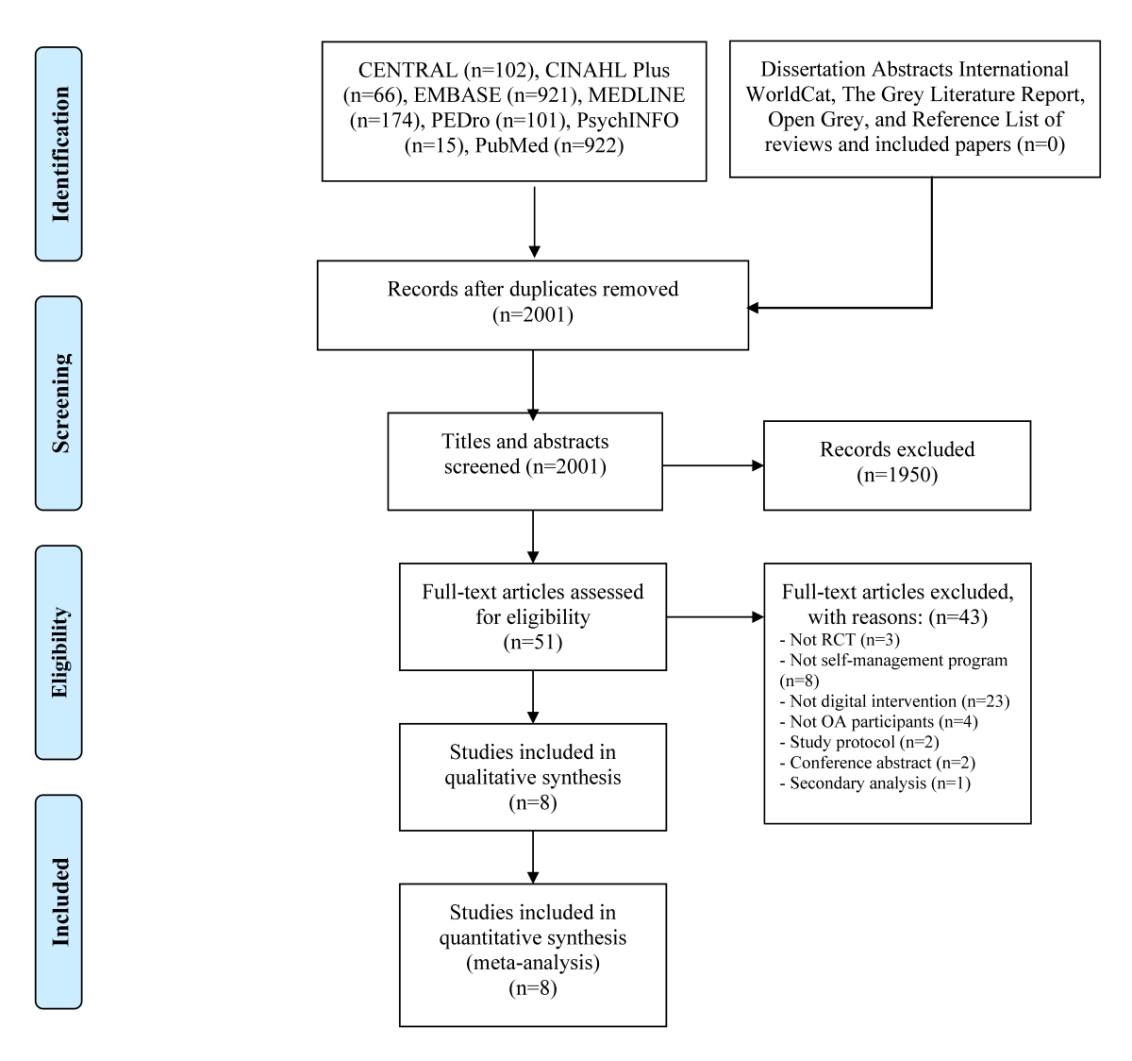
Preferred Reporting Items for Systematic reviews and Meta-Analyses flow diagram. CENTRAL: Cochrane Central Register of Controlled Trials; CINAHL Plus: Cumulative Index to Nursing and Allied Health Literature Plus; EMBASE: Excerpta Medica Database; MEDLINE: Medical Literature Analysis and Retrieval System Online; PEDro Physiotherapy Evidence Database; OA: osteoarthritis; RCT: randomized controlled trial.

A total of 2001 titles and abstracts were screened after excluding duplicates, of which 1950 records did not meet the inclusion criteria (Figure 1). Full texts of 51 potential eligible records were read, and 8 studies published between 2008 and 2018 were included. These studies included 2256 (range 113-352) individuals with OA. A total of 8 digital-based structured SMPs were compared with 10 control conditions. Two studies were cluster RCTs and 6 were RCTs. A list of studies excluded at the full text screening stage with reasons for exclusion is presented in Multimedia Appendix 2 [33-75]. A summary of the study characteristics and participants’ demographics are presented in Tables 1 and 2.

**Table 1:** Characteristics of the included studies.

Author (year)	Country	Participants, n (%)		Intervention type		Outcomes (primary or secondary; baseline mean [SD])		Outcomes (primary or secondary; 95% CI)		Postintervention (weeks); analysis (ITT^a^ or PP^b^)		Follow-up (weeks); analysis (ITT or PP)		Attrition at postintervention, n (%)		
		Experiment	Control	Experiment	Control										Experiment	Control
Allen et al (2010) [76]	United States	172 (33.3)	171 (33.1)	Self-management	TAU^c^		AIMS2^d^: pain (P; 5.9 [2.3]); AIMS2: function (S; 2.6 [1.7])		N/A^e^		52; ITT		NR^f^		26 (15.1)	14 (8.1)
Allen et al (2010) [76]	United States	172 (33.3)	172 (33.3)	Self-management	Health education (attention control)		AIMS2: pain (P; 6.0 [2.3]); AIMS2: function (S; 2.7 [1.8])		N/A		52; ITT		NR		26 (15.1)	14 (8.1)
Allen et al (2016) [77]	United States	151 (50.3)	149 (49.6)	Combined patient and provider OA^g^ management program	TAU		WOMAC^h^: pain (S; 8.5, NR) WOMAC: function (S; 28.7 [NR])		N/A		52; ITT		NR		15 (9.9)	12 (8.1)
Allen et al (2018) [78]	United States	142 (40.5)	68 (19.4)	Internet-based exercise training	WL^i^		WOMAC: pain (S; 6.0 [3.9]); WOMAC: function (S; 21.8 [2.7])		N/A		16; ITT		52; ITT		30 (21.1)	5 (7)
Allen et al (2018) [78]	United States	142 (40.5)	140 (40.0)	Internet- based exercise training	In-person physical therapy		WOMAC: pain (S; 6.1, 3.5); WOMAC: function (S; 22.6 [12.9])		N/A		16; ITT		52; ITT		30 (21.1)	11 (7.8)
Bossen (2013) [79]	The Netherlands	100 (50.2)	99 (49.8)	Join2move: automated web based	WL		N/A		KOOS^j^ and HOOS^k^: function (P; 58.8 [95% CI 51.5-66.0]); KOOS and HOOS: pain (S; 5.4 [95% CI 4.2-6.5]) KOOS and HOOS: QoL (S; 38 [95% CI 30.6-45.5])		12; ITT		52; ITT		16 (16)	15 (15.1)
Kloek (2018) [80]	The Netherlands	109 (50.2)	99 (49.8)	Internet-based exercise	Usual physical therapy		N/A		KOOS and HOOS: function (P; 50.7 [95% CI 45.1-56.4]); KOOS and HOOS: pain (S; 43.9 [95% CI 35.2-52.7]); KOOS and HOOS: QoL^l^ (S; 44.2 [95% CI 38.1-50.4])		12; PP		52; PP		20 (18.3)	(12) 12
Skrepnik (2017) [81]	United States	107 (50.7)	104 (49.3)	Mobile app (OA GO)	TAU		Numeric Pain Rating Scale: (NR; 4.6 [2.3]); 6-min walk test (NR; 402.8 [120.5])		N/A		12; ITT		NR		6 (5.06)	2 (1.9)
Lorig (2008) [82]	United States	433 (50.6)	422 (49.4)	Web-based arthritis self-management program	TAU		Activity Limitation Scale (S; 2.20 [1.03]); Health Assessment Questionnaire; Disability (S; 0.552 [0.402]); Numeric Pain Rating Scale (S; 6.53 [2.23])		N/A		24; ITT and PP		52; ITT and PP		NR	NR
Rini (2015) [83]	United States	58 (51.3)	55 (48.6)	Pain COACH group	No intervention		AIMS2: pain (P; 4.82 [1.73]); AIMS2: function (S; 1.70 [1.30])		N/A		9-12; ITT		NR		1 (1.7)	3 (5.4)

^a^ITT: intention-to-treat.

^b^PP: per protocol.

^c^TAU: treatment as usual.

^d^AIMS: Arthritis Impact Measurement Scales.

^e^N/A: not applicable.

^f^NR: not reported.

^g^OA: osteoarthritis.

^h^WOMAC: The Western Ontario and McMaster Universities Osteoarthritis Index.

^i^WL: waiting list.

^j^KOOS: Knee Injury and Osteoarthritis Outcome Score.

^k^HOOS: Hip disability and Osteoarthritis Outcome Score.

^l^QoL: quality of life.

**Table 2:** Study participants’ demographics.

Author (year), Study arms		Age (years), mean (SD)	Female, n (%)	Affected joint, n (%)			Time since diagnosis (years), mean (SD)
				Knee	Hip	Both	
**Allen et al (2010) [76]**							
	Self-management	60.3 (10.03)	NR^a^ (9)	NR (82)	NR (12)	NR (6)	16.5 (12.7)
	TAU^b^	59.7 (10.1)	NR (6)	NR (79)	NR (17)	NR (4)	15.9 (11.9)
	Health education (attention control)	60.3 (10.8)	NR (7)	NR (79)	NR (16)	NR (5)	15.8 (12.0)
**Allen et al (2016) [77]**							
	Combined patient and provider OA^c^ management program	61.7 (9.0)	20 (13.2)	114 (75.5)	18 (11.9)	19 (12.6)	13.8 (11.1)
	TAU	60.4 (9.4)	8 (5.4)	124 (83.2)	14 (9.4)	11 (7.4)	14.6 (12.1)
**Allen et al (2018) [78]**							
	Internet-based exercise training	65.3 (11.5)	98 (69)	142 (100)	N/A^d^	N/A	11.6 (11)
	Waiting list	64.3 (12.2)	53 (78)	68 (100)	N/A	N/A	14.2 (13)
	In-person physical therapy	65.7 (10.3)	100 (71.4)	140 (100)	N/A	N/A	14.1 (11.6)
**Bossen et al (2013) [79]**							
	Join2move: automated web based	61 (5.9)	60 (60)	67 (67.0)	21 (21.0)	12 (12.0)	NR
	Waiting list	63 (5.4)	69 (70)	60 (60.6)	20 (20.2)	19 (19.2)	NR
**Kloek et al (2018) [80]**							
	Internet-based exercise	63.8 (8.5)	74 (67.9)	71 (65.1)	21 (19.3)	17 (15.6)	NR
	Usual physical therapy	63.3 (8.9)	67 (67.7)	67 (67)	17 (17)	15 (15)	NR
**Skrepnik et al (2017) [81]**							
	Mobile app (OA GO)	61.6 (9.5)	59 (55.1)	100	N/A	N/A	NR
	TAU	63.6 (9.3)	47 (45.2)	100	N/A	N/A	NR
**Lorig et al (2008) [82]**							
	Web-based arthritis self-management program	52.5 (12.2)	NR (90.5)	NR	NR	NR	NR
	TAU	52.2 (10.9)	NR (89.8)	NR	NR	NR	NR
**Rini et al (2015) [83]**							
	Pain COACH group	68.52 (7.65)	46 (79)	18 (33)	9 (16)	28 (51)	NR
	No intervention	66.67 (11.02)	45 (82)	22 (38)	5 (9)	31 (53)	NR

^a^NR: not reported.

^b^TAU: treatment as usual.

^c^OA: osteoarthritis.

^d^N/A: not applicable.

### Participants

A total of 6 studies were conducted in the United States, and 2 were performed in the Netherlands.

The number of participants in the studies ranged from 113 to 855. The median (IQR) age was 63 (3.55) years. Most of the participants in the 6 studies were female (median 74.7%, IQR 6.9%), whereas in the remaining 2 studies, most participants were men (93.0% and 90.7%).

Two studies recorded participants with knee OA only; 5 studies recorded a mixture of knee (median 71.55%, IQR 15.77%), hip (median 16%, IQR 5.75%), or both (median 6%, IQR 10.5%); and 1 study did not record the area affected. No studies recorded OA in the hand or any other type of OA. Five of the studies recorded the time since diagnosis, with a median of 15 (IQR 2) years.

### Intervention Groups

#### Intervention Delivery Mechanisms

Out of the studies reviewed, 2 used telephone, audio and video, and written materials to deliver an SMP derived from social cognitive theory and 5 studies delivered an internet-based exercise training: 1 study was delivered by a physical therapist, 2 studies were delivered in a combination of face-to-face time with a physical therapist, 1 study had a virtual coach, and 1 study was self-administered only. The remaining study used a mobile app with a wearable monitor in combination with a physician face-to-face. Three studies delivered interventions over 52 weeks, 2 studies had 12-week interventions, and 1 study each had interventions lasting 6, 8, and 9 weeks (Table 3).

**Table 3:** Detailed intervention delivery mechanisms.

Author (year)	Medium or method (Y or N)^a^							Professional input or support		Timing			Tailoring (Y or N)		Modification (Y or N)		Adherence assessed: Y or N; Completion: n (%)	
	Telephone	Audio and/or video	Internet	Mobile app	Wearables	Written material and/or booklet	Face-to-face element				SMP^b^ delivery period (weeks)	Number of support sessions						
Allen et al (2010) [76]	Y	Y	N	N	N	Y	N		Telephone calls by health educator		52	Once a month for 12 months		Y		N		Y; NR^c^
Allen et al (2016)^d^ [77]	Y	Y	N	N	N	Y	N		Telephone calls by counselor trained in OA^e^ and behavior change		52	Twice a week for 6 weeks plus once a week for 6 weeks		Y		N		N; NR
Allen et al (2018) [78]	N	N	Y	N	N	N	N		Physical therapist administered the intervention		52	Up to 8		Y		N		Y; 114 (80.2)
Bossen (2013) [79]	N	N	Y	N	N	N	N		None		9	N/A^f^		Y		Y		Y; 46 (46)
Kloek et al (2018) [80]	N	N	Y	N	N	N	Y		Face-to-face with physical therapist		12	5 over 12 weeks		N		Y		Y; NR (81)
Skrepnik et al (2017) [81]	N	N	N	Y	Y	N	Y		Face-to-face with physician investigator plus trial coordinator demonstrated the app		12	5 over 12 weeks		Y		N		Y; 90 (82.5)
Lorig et al (2008) [82]	N	N	Y	N	N	Y	N		SMP-trained moderator facilitating the program		6 weeks	NR		Y		N		Y; approximately 95%^g^
Rini et al (2015) [83]	N	N	Y	N	N	Y	N		Virtual coach led participants through the program		8	Once a week for 8 weeks		N		N		Y; 53 (91)

^a^Y: yes and N: no.

^b^SMP: self-management program.

^c^NR: not reported.

^d^The intervention also included P*rovider Intervention*, which involved delivery of patient-specific recommendations at the point of care.

^e^OA: osteoarthritis.

^f^N/A: not applicable.

^g^Logged at least once into the program.

#### Self-Management Components

All studies used health education as a component of self-management. For 5 studies, this was the main component. Additional self-management components included goal setting (n=6); action planning (n=4); and exercise components such as physical activity (n=6), aerobic (n=5), resistive (n=4), flexibility (n=3), and balance (n=1) were recorded.

Additional self-management components included diet and/or weight management (n=5); pain management (n=6); medication (n=3); motivation (n=6); peer support (n=2); patient-therapist communication (n=2); and stress management, relaxation, or sleep (n=4; Tables 4 and 5).

A range of theories were used to inform the intervention, including Social Cognition Theory (n=3), Self-Efficacy Theory (n=2), and behavioral graded activity theory (n=2). One study stated that they used a combination of Social Cognition Theory, adult learning theory, and principles of multimedia instruction (Tables 4 and 5).

**Table 4:** Detailed self-management program components.

Author (year)	Education	Goal setting	Action planning	Exercise^a^ components or PA						Exercise dose; (weeks×frequency×minute)
				Aerobic	Resistive	Flexibility	Balance	Physical activity		
Allen et al (2010) [76]	✓^b^	✓^b^	✓^b^	NR^c^	NR	NR	NR	✓^d^	NR	
Allen et al (2016)^e^ [77]	✓^d^	✓^b^	✓^b^	✓^d^	✓^d^	✓^d^	—	✓^d^	52×2×75	
Allen et al (2018) [78]	✓^d^	—	✓^d^	✓^b^	✓^b^	✓^b^	—	—	Aerobic: 52×7×NR; resistive and flexibility: 52×3×NR	
Bossen et al (2013) [79]	✓^b^	✓^b^	—	✓^d^	—	—	—	✓^b^	Varied with gradual increments	
Kloek et al (2018) [80]	✓^b^	—	—	✓^d^	✓^b^	—	✓^b^	✓^b^	12×3×NR (with gradual increments)	
Skrepnik et al (2017) [81]	✓^d^	✓^b^	—	N/A^f^	N/A	N/A	N/A	✓^b^	12×7×NR	
Lorig et al (2008) [82]	✓^b^	✓^d^	✓^b^	✓^d^	✓^d^	✓^d^	—	—	Varied: tailored to individual	
Rini et al (2015) [83]	✓^b^	✓^d^	—	N/A	N/A	N/A	N/A	✓^d^	NR	

^a^All studies had exercise as part of the intervention, except Skrepnik et al [81] and Rini et al [83].

^b^Main components of the intervention.

^c^NR: not reported.

^d^Other components of the intervention.

^e^The intervention also included *Provider Intervention*, which involved delivery of patient-specific recommendations at the point of care.

^f^N/A: not applicable.

**Table 5:** Detailed self-management program components (continued).

Author (Year)	Diet or weight management		Pain management		Medication		Motivation		Peer support		Patient-therapist communication		Stress management, relaxation and/or sleep		Theory	
Allen et al (2010) [76]	✓^a^		✓^a^		✓^a^		—^b^		—		✓^a^		✓^a^		SCT^c^	
Allen et al (2016)^d^ [77]	✓^a^		✓^a^		—		✓^a^		—		—		✓^a^		SCT	
Allen et al (2018) [78]	✓^a^		✓^a^		—				—		—		—		SET^e^	
Bossen et al (2013) [79]	—		—		—		✓^a^		—		—		—		BGAT^f^	
Kloek et al (2018) [80]	✓^a^		✓^a^		✓^a^		✓^a^		—		—		—		BGAT	
Skrepnik et al (2017) [81]	—		—		—		✓^a^		—		—		—		NR^g^	
Lorig et al (2008) [82]	✓^a^		✓^h^		✓^a^		✓^a^		✓^h^		✓^a^		✓^a^		SET	
Rini et al (2015) [83]	—		✓^h^		N/A^i^		✓^a^		✓^a^		—		✓^h^		SCT, ALT^j^, PMI^k^	

^a^Other components of the intervention.

^b^Components not included in the intervention.

^c^SCT: social cognitive theory.

^d^The intervention also included *Provider Intervention*, which involved delivery of patient-specific recommendations at the point of care.

^e^SET: self-efficacy theory.

^f^BGAT: Behavior Graded Activity Theory.

^g^NR: not reported.

^h^Main components of the intervention.

^i^N/A: not applicable.

^j^ALT: adult learning theory.

^k^PMI: principles of multimedia instruction.

#### Behavior Change Components

A variety of behavior change techniques have been used. The most common techniques involved goal setting and planning (n=8), feedback and monitoring (n=7), and shaping knowledge (n=7). All studies used at least four different groups of techniques, with Rini et al [83] using 10 group techniques [29] (Table 6; Multimedia Appendix 3). Owing to the heterogeneity in the approaches used, a meta-analysis was not appropriate.

**Table 6:** Behavior change techniques used within included studies.

Author (year)	BCT^a^ taxonomy grouping^b^															
	1^c^	2^d^	3^e^	4^f^	5^g^	6^h^	7^i^	8^j^	9^k^	10^l^	11^m^	12^n^	13^o^	14^p^	15^q^	16^r^
Allen et al (2010) [76]	1.1^s^, 1.2^s^, 1.4^s^	—^t^	3.1^s^	4.1^s^, 4.2^s^	5.1^s^	—	—	—	—	—	—	—	—	—	—	—
Allen et al (2016) [77]	1.1, 1.4, 1.4, 1.5 ^s^	2.3^s^	3.1, 3.2^s^, 3.3^s^	4.1	5.1^s^	6.1^s^	—	8.1^s^	—	—	—	—	—	—	—	16.2^s^
Allen et al (2018) [78]	1.7^s^	2.3, 2.4^s^	3.2	4.1, 4.2	5.1	6.1	—	8.7^s^	—	—	—	—	—	—	—	—
Bossen et al (2013) [79]	1.1	2.4	3.1	4.1	—	6.1	—	8.7	—	—	—	—	—	—	15.1 ^s^	—
Kloek et al (2018) [80]	1.1	2.3, 2.6^s^	3.1, 3.2	4.1, 4.2	—	6.1	—	8.7	—	—	—	—	—	—	—	—
Skrepnik et al (2017) [81]	1.1, 1.5	2.2^s^, 2.4,2.6	—	—	5.1, 5.4^s^	—	7.1^s^	—	—	—	—	—	—	—	—	—
Lorig et al (2008) [82]	1.2, 1.4	2.2, 2.3	3.2	4.1	—	—	—	—	—	—	11.2^s^	12.6^s^	—	—	15.4^s^	16.3^s^
Rini et al (2015) [83]	1.1, 1.2, 1.4, 1.5	2.2, 2.4	—	4.1	5.4 5.5^s^	6.2^s^, 6.3^s^	—	8.1, 8.3^s^	9.2^s^	—	11.2	12.4^s^	—	—	15.1	—

^a^BCT: behavior change technique.

^b^Multimedia Appendix 3 provides an explanation of the grouping and example text from study papers.

^c^1: Goals and planning

^d^2: Feedback and monitoring

^e^3: Social support

^f^4: Shaping knowledge

^g^5: Natural consequences

^h^6: Comparison of behavior

^i^7: Associations

^j^8: Repitition and substitution

^k^9: Comparison of outcomes

^l^10: Rewards and threats

^m^11: Regulation

^n^12: Antecedents

^o^13: Identity

^p^14: Scheduled consequences

^q^15: Self-belief

^r^16: Covert learning

^s^1.1: goal setting (behavior); 1.2: problem-solving; 1.4: action planning; 1.5: review behavior goal(s); 1.7: review outcome goal(s); 2.2: feedback on behavior; 2.3: self-monitoring of behavior; 2.4: self-monitoring of outcome(s) of behavior; 2.6: biofeedback; 3.1: social support (unspecified); 3.2: social support (practical); 3.3: social support (emotional); 4.1: instruction on how to perform the behavior; 4.2: information about antecedents; 5.1: information about health consequences; 5.4: monitoring of emotional consequences; 5.5: anticipated regret; 6.1: demonstration of the behavior; 6.2: social comparison; 6.3: information about others’ approval; 7.1: prompts or cues; 11.2: reduce negative emotions; 12.4: distraction; 12.6: body changes; 15.1: verbal persuasion about capability; 15.4: self-talk; 16.2: imaginary reward; 16.3: vicarious consequences.

^t^Behavior change techniques not used in the study.

### Comparison

Digital self-management interventions were compared with treatment as usual (n=4), wait list control (n=2), in-person physical therapy (n=2), attention group (health education; n=1), and no intervention (n=1).

### Risk of Bias

**Figure 2:**
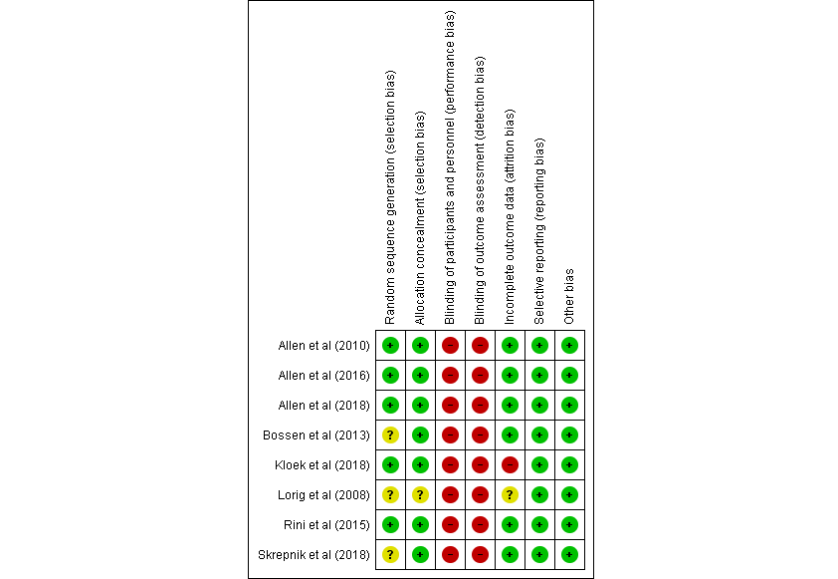
Summary of risk of bias assessment.

The risk of bias was assessed as unclear for random sequence generation (n=3), allocation concealment (n=1), and incomplete outcome data (n=1). There was a high risk of bias for incomplete outcome data in 1 study (Figure 2). The detection bias and performance bias were rated high in all studies because of the nature of the interventions and patient-reported outcome tools.

### Effects of Interventions

A summary of findings for the main comparisons of the study with GRADE ratings is presented in Table 7. Detailed meta-analytic forest plots are also presented in Multimedia Appendix 4.

**Table 7:** Summary of findings and Grading of Recommendations, Assessment, Development, and Evaluations ratings for the main comparisons.

Outcomes	Number of participants (studies)	Quality of the evidence^a^ (GRADE^b^)	Comments	Illustrative comparative risks (95% CI)	
				Assumed risk control	Corresponding risk intervention (95% CI)
Pain (D-SMP^c^ vs TAU^d^ control)**:** AIMS2^e^, WOMAC^f^, HOOS^g^/KOOS^h^, NPRS^i^; follow-up: 9-52 weeks	1614 (7 studies)	⊕⊕⊕⊝ Moderate^j^	Limitation (–1)	No risk assumed	The mean pain in the intervention groups was 0.28 SDs lower (0.38-0.18 lower)
Pain (D-SMP vs TAU control)**:** WOMAC, HOOS/KOOS, NRS PAIN; follow-up: ≥52 weeks	716 (3 studies)	⊕⊕⊕⊝ Moderate^j^	Limitation (–1)	No risk assumed	The mean pain in the intervention groups was 0.20 SDs lower (0.35-0.05 lower)
Pain (internet-SMP vs physical therapy)**:** WOMAC, HOOS/KOOS; follow-up: 12-52 weeks	456 (2 studies)	⊕⊕⊝⊝ Low^j,k^	Limitation (–1); imprecision (–1)	No risk assumed	The mean pain in the intervention groups was 0.07 SDs lower (0.25 lower to 0.01 higher)
Pain (internet-SMP vs physical therapy)**:** WOMAC, HOOS/KOOS; follow-up: ≥52 weeks	416 (2 studies)	⊕⊕⊝⊝ Low^j,k^	Limitation (–1); imprecision (-1)	No risk assumed	The mean pain in the intervention groups was 0.12 SDs lower (0.31 lower to 0.07 higher)
Pain (telephone- and video-SMP vs attention control); AIMSS2	306 (1 study)	⊕⊕⊝⊝ Low^l^	Unknown consistency (–2)	No risk assumed	The mean pain in the intervention groups was 0.26 SDs lower (0.49 lower to 0.04 lower)
Physical function (D-SMP vs TAU: AIMS2, WOMAC, HOOS/KOOS, 6MWT^m^, ALS^n^; follow-up: 9-52 weeks	1625 (7 studies)	⊕⊕⊕⊝ Moderate^j^	Limitation (-1)	No risk assumed	The mean physical function in the intervention groups was 0.26 SDs higher (0.35-0.16 higher)
Physical function (D-SMP vs TAU control): WOMAC, KOOS/HOOS, ALS; follow-up: ≥52 weeks	707 (3 studies)	⊕⊕⊕⊝ Moderate^j^	Limitation (–1)	No risk assumed	The mean pain in the intervention groups was 0.23 SDs higher (0.38-0.08 higher)
Physical function (internet-SMP vs active control): WOMAC, HOOS/KOOS follow-up: 12-52 weeks	258 (2 studies)	⊕⊕⊝⊝ Low^j,k^	Limitation (–1); imprecision (–1)	No risk assumed	The mean physical function in the intervention groups was 0.05 SDs lower (0.13 higher to 0.23 lower)
Physical function (internet-SMP vs active control)**:** WOMAC, HOOS/KOOS; follow-up: ≥52 weeks	416 (2 studies)	⊕⊕⊝⊝ Low^j,k^	Limitation (–1); imprecision (–1)	No risk assumed	The mean physical function in the intervention groups was 0.03 SDs higher (0.22 higher to 0.16 lower)
Physical function (telephone- and video-SMP vs attention control)**:** AIMS2	306 (1 study)	⊕⊝⊝⊝ Very low^k,l^	Imprecision (–1); unknown consistency (–2)	No risk assumed	The mean physical function in the intervention groups was 0.17 SDs higher (0.39 higher to 0.06 lower)
Disability (internet-SMP vs TAU): HAQ^o^	352 (1 study)	⊕⊝⊝⊝ Very low^j,l^	Limitation (–1); unknown consistency (–2)	No risk assumed	The mean disability in the intervention groups was 0.10 SDs lower (0.17-0.03 lower)
Quality of life (internet-SMP vs TAU): HOOS/KOOS	165 (1 study)	⊕⊝⊝⊝ Very low^j,k,l^	Imprecision (–1); unknown consistency (–2)	No risk assumed	The mean quality of life in the intervention groups was 0.17 SDs higher (0.47 higher to 0.14 lower)

^a^GRADE Working Group grades of evidence: High quality: further research is very unlikely to change our confidence in the estimate of effect; Moderate quality: further research is likely to have an important impact on our confidence in the estimate of effect and may change the estimate; Low quality: further research is very likely to have an important impact on our confidence in the estimate of effect and is likely to change the estimate; Very low quality: we are very uncertain about the estimate.

^b^GRADE: Grading of Recommendations, Assessment, Development, and Evaluations.

^c^D-SMP: digital-based structured self-management program.

^d^TAU: treatment as usual.

^e^AIMS: Arthritis Impact Measurement Scales.

^f^WOMAC: Western Ontario and McMaster Universities Osteoarthritis Index.

^g^HOOS: Hip disability and Osteoarthritis Outcome Score.

^h^KOOS: Knee Injury and Osteoarthritis Outcome Score.

^i^NPRS: numerical pain rating scale.

^j^Majority of the evidence comes from studies with unclear randomization and/or allocation concealment.

^k^Total sample size is small. Total effect size has CIs crossing the no effect line.

^l^Unknown consistency and/or publication bias.

^m^6MWT: 6-minute walk test.

^n^ALS: Activity Limitation Scale.

^o^HAQ: Health Assessment Questionnaire.

#### Main Outcomes

##### Pain

Seven studies involving 1614 participants reported the effect of digital-based structured SMPs compared with usual care control condition on pain outcome at the postintervention time point (range 9-52 weeks). Overall, a significant and medium effect in favor of the intervention was observed on pain reduction (SMD –0.28; 95% CI –0.38 to –0.18); heterogeneity (X^2^_6_=5.1; *P*=.53; I²=0%). Three studies with 716 participants reported a long-term (≥12 months) effect of digital-based structured self-management program compared with usual care conditions, indicating a significant and small overall effect (SMD –0.20; 95% CI –0.35 to –0.05); heterogeneity (X^2^_2_=0.4; *P*=.83; I²=0%). Using the GRADE approach, the quality of evidence was rated *moderate* for both postintervention and long-term follow-up comparisons because of the high risk of bias in most studies (ie, study limitations).

Two studies with 456 participants comparing digital-based structured SMPs (internet) and physical therapy did not show a difference in pain reduction between the 2 intervention conditions (SMD –0.07; 95% CI –0.25 to 0.11); heterogeneity (X^2^_1_=0.2; *P*=.64; I²=0%). The effect of the digital-based structured SMP slightly increased at the 12-month follow-up time point but was not significant (SMD –0.12; 95% CI – 0.31 to 0.07; heterogeneity X^2^_1_=0.1; *P*=.94; I²=0%). The quality of evidence for both time points was rated as *low* because of the high risk of bias (ie, study limitation) and total small sample size (ie, imprecision).

The only study comparing digital-based structured SMPs with attention control (health education) condition [76] (n=306 participants) showed a medium and significant effect on pain reduction in favor of digital-based structured SMPs (SMD –0.26; 95% CI –0.49 to –0.04). The quality of evidence was rated as *low* because of unknown consistency and/or publication bias.

##### Physical Function

A total of 7 studies involving 1625 people with OA reported the effect of digital-based structured SMPs compared with usual care control conditions on patient-reported physical function outcomes at the postintervention time point (range 9-52 weeks). Overall effect size indicates a medium and significant improvement in function in favor of the SMP (SMD –0.26; 95% CI –0.35 to –0.16; heterogeneity X^2^_6_=5.2; *P*=.52; I²=0%). Studies reporting the 12-month follow-up data (n=3 studies; n=707 participants) showed medium and significant overall effect of the digital-based structured SMP compared with usual care control condition (SMD –0.23; 95% CI –0.38 to –0.08; heterogeneity X^2^_2_=0.1; *P*=.96; I²=0%). The quality of evidence was rated as *moderate* for both comparisons because of the high risk of bias in most studies (ie, study limitation).

The 2 studies comparing digital-based structured SMPs (internet) with physical activity did not show a significant difference between the 2 interventions neither at postintervention nor at longer follow-up time points (SMD 0.05; 95% CI –0.13 to 0.23; heterogeneity X^2^_1_=0.1; *P*=.77; I²=0%; and SMD –0.03; 95% CI –0.22 to 0.16; heterogeneity X^2^_1_=0.01; *P*=.90; I²=0%, respectively). The quality of evidence was rated *low* for both time points due to *limitation* and *imprecision*.

The digital-based structured SMP versus attention control group (health education), compared in 1 study [76], resulted in a small and nonsignificant (*P*=.15) improvement in physical function (SMD –0.17; 95% CI –0.39 to 0.06). The quality of evidence for this intervention was rated *very low* because of *imprecision* (ie, small sample size) and unknown consistency and/or publication bias.

##### Disability

One study [82] reported disability measures in 352 participants. The results show a minimal effect size in favor of internet-based SMPs compared with the usual care condition (SMD –0.10; 95% CI –0.17 to –0.03). However, the effect of intervention did not persist after 12 months of follow-up (SMD –0.00; 95% CI –0.21 to 0.20). The quality of evidence for this outcome at postintervention was rated as *very low* because of the high risk of bias in the study and unknown consistency and/or publication bias. The quality of evidence for the outcome at the 12-month follow-up was rated *very low* because of *imprecision* (ie, small sample size) and unknown consistency and/or publication bias for the follow-up time point.

##### Quality of Life

Only 1 study [79] reported the QoL outcome measure in 165 people with OA, indicating that the internet-based SMP did not make a significant improvement in QoL neither at 4 months postintervention nor at the 12-month follow-up (SMD –0.17; 95% CI –0.47 to 0.14 and SMD –0.07; 95% CI –0.39 to 0.26, respectively). The quality of evidence for this intervention at both time points was rated *very low* because of *imprecision* (ie, small sample size) and unknown consistency and/or publication bias.

##### Cost and Resource Use

Data reported on the cost of these interventions and resources used are limited. Allen et al [76] calculated cost by excluding nonrecurring labor costs, such as time for training a health educator at salary cost and the making of the intervention. The cost calculated included labor intervention delivery costs and indirect nonlabor costs, such as printing educational materials and creating compact discs. It was reported that the per-participant costs were US $107 (range US $100-US $121) for OA self-management and US $51 (range US $47-US $60) for health education. This cost was not compared with that of usual care. In the study by Lorig et al [82], no significant difference was reported between digital-based structured SMPs and usual care in terms of health care utilization (ie, physician, emergency, chiropractic or physical therapists visits or days in hospital).

### Subgroup Analysis

The planned subgroup analysis based on the digital medium used to deliver the intervention revealed that the mobile app (n=1 study, n=197 participants) resulted in the largest effect size in pain reduction (SMD –0.38; 95% CI –0.67 to –0.10), followed by the internet medium (n=4 studies, n=841 participants; SMD –0.33; 95% CI –0.46 to –0.19; I²=0%). The telephone as a medium of intervention delivery resulted in the smallest effect size in pain reduction (n=2 studies, n=576 participants; SMD –0.18; 95% CI –0.35 to –0.02; I²=0%). However, the difference between the 2 groups was not significant (X^2^_2_=2.3; *P*=.32; I²=13.4%). Subgroup differences for physical function outcome were not significant (X^2^_2_=0.1; *P*=.97; I²=0%) between telephone, internet, and mobile apps, and resulted in similar small effect sizes (SMD –0.23; 95% CI –0.40 to –0.07, SMD –0.27; 95% CI –0.41 to –0.14, and SMD –0.24; 95% CI –0.51 to 0.03, respectively).

The intervention in most studies had exercise and/or physical activity components, and, in a few studies, it was the main component of the intervention. Therefore, we conducted a subgroup analysis accordingly. The pain reduction in studies with exercise and/or physical activity as the main component of the intervention was greater than the pain reduction in those studies without exercise and/or physical activity as the main component of the intervention (SMD –0.37; 95% CI – 0.54 to –0.20 and SMD –0.23; 95% CI –0.36 to –0.11, respectively). However, the difference was not statistically significant (X^2^_1_=1.7; *P*=.19; I²=40.6%). The improvement in physical function was comparable in both groups—(SMD –0.27; 95% CI – 0.44 to –0.11) in studies with exercise and/or physical function as the main component and (SMD –0.24; 95% CI –0.38 to –0.11) in studies without exercise as the main component (X^2^_1_=0.1; *P*=.76; I²=0%).

No subgroup analysis could be conducted for the follow-up time points as all 3 studies reporting the 12-month follow-up data used the internet as the intervention delivery medium. Similarly, no subgroup analysis could be conducted on the studies with high rates of completion because intervention was delivered via the telephone in 2 studies that did not report completion rate, or there was no measure of adherence.

### Risk of Bias Sensitivity Analysis

Risk of bias sensitivity analysis, excluding studies with no or unclear random allocation, inadequate treatment allocation concealment, and/or incomplete outcome data, reduced the effect size in both pain reduction and improvement in physical function (SMD –0.19; 95% CI –0.32 to –0.05 and SMD –0.19; 95% CI –0.32 to –0.06, respectively).

## Discussion

### Principal Findings

The findings of this review indicate that digital-based structured SMPs compared with the treatment as usual or no intervention control groups resulted in a significant, homogeneous, and medium reduction in pain (SMD –0.28; 95% CI –0.38 to –0.18) and improvement in physical function (SMD –0.26; 95% CI –0.35 to –0.16). The SMDs reduced slightly at longer follow-up time points but remained significant with medium effect sizes. However, the findings should be interpreted with caution as the overall quality of the body of evidence is moderate because of the risk of bias in the included studies. Using SD from the control group of the largest (most adequately powered) study by Lorig et al [82], the SMDs translate to 5.70% reduction in pain and 5.07% improvement in physical function at postintervention time points. In accordance with Tubach et al [84] and Angst et al [85], we determined a minimal clinically important difference (MCID) of 15% in pain and 8% in physical function. Therefore, these effects are unlikely to be clinically significant. The findings on disability and QoL are less clear, as these outcomes were reported in 1 study with very small effect sizes.

Both immediate and longer-term effects of digital-based structured SMPs on pain in our review (SMD –0.28; 95% CI –0.38 to –0.18 and SMD –0.20; 95% CI –0.35 to –0.5, respectively) were slightly greater than those reported in a Cochrane review comparing standard OA SMPs with usual care (SMD –0.26; 95% CI –0.41 to –0.10 and SMD –0.17; 95% CI –0.26 to –0.08, respectively) [19]. However, the immediate and longer-term effect of digital-based structured SMPs on physical function was greater in our review compared with the immediate effect of SMPs reported in the Cochrane review by Kroon et al (SMD 0.01; 95% CI –019 to 0.18 and SMD –0.16; 95% CI –0.25 to –0.01, respectively) [19]. Despite the moderate quality of evidence reported in both reviews and a lack of direct comparison between the digital-based structured SMP and the SMP intervention, it could be postulated that the digital-based structured SMPs may have similar or greater effects than conventional SMPs on pain and physical function. Considering the potential cost-effectiveness of digital interventions, digital-based structured SMPs have the potential to reach populations reluctant or unable to attend face-to-face appointments. This could have a considerable benefit from a public health perspective despite the modest size of the effect.

Previous reviews indicate that eHealth interventions have the potential to reduce treatment costs [21,86]. We could not assess intervention costs because none of the included studies compared intervention costs across groups. In the study by Kloek et al [80], participants in the intervention group (internet-based exercise) had 5 in-person physical therapy sessions, whereas those in the control group visited a physical therapist an average of 12 times. Nevertheless, both groups showed a significant improvement in most health outcomes. Reduced numbers of physical therapy visits would likely result in a reduction in health care costs. Future RCTs of digital-based structured SMPs are encouraged to assess the cost-effectiveness of digital interventions in isolation or combination with face-to-face sessions. It should be noted that smartphones and/or internet devices are now owned by the majority of the population; therefore, these interventions are accessible and can be delivered conveniently and easily to the target audience. However, it should be noted that digital-based structured SMPs may not be suitable for all patients, potentially because of age, preference, comorbidities, and/or severity of illness; therefore, the intervention must be tailored to the needs, preferences, and conditions of patients and include face-to-face, group, and digital modes in the intervention package.

The result of the subgroup analysis indicates that there is no significant difference among the different digital modes of SMP delivery. However, mobile app (1 study) and internet SMPs (4 studies) resulted in medium effects on pain outcome, whereas telephone SMPs (2 studies) showed a small effect. Potential reasons for this observation could be that the majority of participants in the telephone studies were male veterans (>90%) who had more comorbidities and/or severe OA symptoms. In addition, the interventions were low intensity, and telephone call sessions were short (average 16.6 min). However, the small number of studies and the fact that the 2 telephone trials had a low risk of bias cautions against this interpretation; inflated effect size in studies with a high risk of bias is expected. The results of further subgroup analysis indicate that studies with exercise or physical activity as the main component of the intervention resulted in a greater improvement in pain but not in perceived physical function. A possible explanation for the reduced pain in this group of studies could be because of the change in pain tolerance and decreased perception of pain after exercise [87,88]. Four studies (n=2 telephone and n=2 internet) were included in the risk of bias sensitivity analysis. The analysis resulted in a reduction in the effect size compared with the main meta-analysis for both pain and physical function outcomes. Notably, the 2 combined telephone and video studies contributed most to the SMD (weight >65%) in the sensitivity analysis.

Attrition at postintervention was modest; median 10% (IQR 7%) in the intervention groups and median 8% (IQR 4%) in the control conditions. Despite common concerns with high dropout rates in digital interventions [89], the reported attrition rates in the included studies appear reasonable compared with behavioral studies. In a recent study, Bennell et al [90] reported that a web-based exercise program improved home exercise adherence and confidence in the ability to undertake exercise compared with a home-based exercise program prescribed by a physical therapist’s usual methods [90]. In our review, treatment adherence in 6 studies reporting treatment completers was 46% in 1 study [79], >80% in 3 studies [78,80,81], and >90% in 2 studies (Table 1) [82,83]. There are a few possible reasons for the relatively low dropout and nonadherence rates. First, interventions in all studies were tailored to participants’ needs and conditions. Second, researchers used features in web-based or mobile apps to develop reminder and monitoring systems and created higher interactivity of the intervention delivery. Third, in some studies, health professionals maintained contact with the study participants during the study through either face-to-face meetings or telephone calls. However, it should be noted that the participants were highly educated in most studies and, in some studies, self-selected (ie, responded to the study participants’ recruitment advertisement). Thus, they may have been enthusiastic about the new intervention. In future research and development, the advanced features of mobile apps and internet interventions could be employed to deliver even more effective and tailored monitoring and/or motivational interventions. Digital interventions can also be used to show patients’ symptom improvement, which is a useful and effective way not only to improve retention and compliance but also to increase their effect through self-efficacy [91,92].

There are a few limitations to the current review. First, only studies published in English were considered. Second, blinding of intervention and outcome assessment is not possible; therefore, all studies suffer from performance and detection biases. Moreover, some of the most powered studies have a high risk of bias, lowering the overall quality of evidence. Third, disability and QoL outcomes were only reported in 1 study each. Fourth, this review has limited generalizability because participants in most studies were highly educated and self-selected; thus, they may have been highly motivated. Finally, interventions in some studies were targeted to exercise and/or physical activity as the main component of the program, and behavior change techniques were used to improve exercise adherence [78-81]; the multiplicity and heterogeneity of techniques prohibited meta-analysis of the effects of particular techniques. However, almost all studies employed theoretically driven interventions incorporating important components of the SMPs (ie, education, goal setting, action planning, problem solving, skills acquisition, self-monitoring, understanding illness, and managing emotions).

In conclusion, digital-based structured SMPs resulted in medium improvements in pain and physical function postintervention. However, the effects are below the MCID and may, therefore, not be clinically significant. The quality of the evidence for pain and function was graded as *medium* because of the high risk of bias in the studies; therefore, the true effect is likely to be close to our estimate of the effect, but there is a possibility that it is substantially different. The effect of digital-based structured SMP intervention on pain and physical function was slightly reduced at the 12-month follow-up, but remained at the medium threshold. One study reporting results on disability and one study reporting results on health-related QoL indicated small improvements in both outcomes. We rated the quality of the evidence as *low* and *very low* for disability and health-related QoL, respectively, indicating that the true effects are likely to be substantially different from our estimate of effect.

### Conclusions

This review of digital-based structured SMPs on self-reported outcomes including pain, physical function, disability, and health-related QoL in patients with OA revealed 6 RCTs and 2 cluster RCTs. digital-based structured SMPs resulted in medium improvements in pain and physical function postintervention, but these may not be clinically significant. These effects were slightly reduced at the 12-month follow-up, but remained at the medium threshold. The quality of the evidence for pain and function was graded as *medium* because of a high risk of bias in some studies. More high-quality studies are needed, and the routine assessment of QoL and disability would be useful.

## Appendix

Multimedia Appendix 1 Search strategy.

Multimedia Appendix 2 Studies excluded with reasons.

Multimedia Appendix 3 Behavior change technique taxonomy grouping with explanation and example texts from studies.

Multimedia Appendix 4 Forest plots of review comparisons.

## Abbreviations

eHealth: electronic health
GRADE: Grading of Recommendations, Assessment, Development, and Evaluations
MCID: minimal clinically important difference
NIHR: National Institute for Health Research
OA: osteoarthritis
QoL: quality of life
RCT: randomized controlled trial
SMD: standardized mean difference
SMP: self-management program
TiDieR: template for intervention description and replication
WHO: World Health Organization
