# Supplementary material for: Digital Self-Management Interventions for People With Osteoarthritis: Systematic Review With Meta-Analysis
Source: J Med Internet Res. 2020 Jul 20;22(7):e15365. doi: 10.2196/15365 (PMC7428148; doi:10.2196/15365)
Supplement: Multimedia Appendix 1 [file jmir_v22i7e15365_app1.docx]

# Multimedia Appendix 1: Search Strategy

## MEDLINE

Date searched: 18 May 2018

| **#** | **Searches** | | **Results** |
| --- | --- | --- | --- |
| S1 | (MM "Osteoarthritis+") OR ((degenerative or noninflammatory OR non-inflamatory OR non inflamatory) W2 (Joint OR arthritis)) OR Osteoarthr* | Search modes - Boolean/Phrase | 83,451 |
| S2 | (MM "Self Care+") OR (MM "Education, Nonprofessional+") OR (MM "Patient Education as Topic+") OR ((self or symptom*) W2 (car* or help or manag* or directed or monitor* or efficac* or admin*)) OR ((health or patient*) W2 (educat* or information or counsel* or instruct*)) OR ((management*) N2 (plan or disease*)) OR ((chang*) N2 (behaviour* or behavior*)) | Search modes - Boolean/Phrase | 525,309 |
| S3 | (MM "Telemedicine+") OR (MM "Remote Consultation+") OR (MM "Teleradiology") OR (MM "Telenursing") OR (MM "Computer Systems+") OR (MM "Educational Technology+") OR (MM "Computer-Assisted Instruction") OR (MM "Hypermedia") OR (MM "Video Games") OR (MM "Cell Phones+") OR (MM "Social Networking") OR (MM "Virtual Reality Exposure Therapy") OR ((Internet OR web OR computer) W (base* OR deliver*)) OR telecare OR telerehab* OR telemed* OR telehealth OR teletherap* OR e-health OR m-health OR Ehealth OR Mhealth OR Computer* OR microcomputer* OR PC OR PCs OR Mac OR Macs OR technolog* OR telecommunication OR multi-media OR multimedia* OR hypermedia* OR app OR apps OR Email OR E-mail OR ((cell* OR mobile*) W3 phone*) OR Mobile OR Smartphone OR smart-phone OR smart telephone OR Tablet OR cell OR hand-held OR handheld OR VR OR virtual reality | Search modes - Boolean/Phrase | 6,070,036 |
| S4 | (MM "Randomized Controlled Trial+") OR (MM "Randomized Controlled Trials as Topic+") OR (MM "Cross-Over Studies") OR Randomized controlled trial OR randomized OR randomised OR randomly OR placebo OR clinical OR Cross over OR Crossover OR RCT | Search modes - Boolean/Phrase | 4,344,289 |
| S5 | (MM "Animals+") | Search modes - Boolean/Phrase | 1,065,953 |
| S6 | S4 NOT S5 | Search modes - Boolean/Phrase | 4,286,413 |
| S7 | S1 AND S2 AND S3 AND S6 | Search modes - Boolean/Phrase | 179 |
| S8 | S1 AND S2 AND S3 AND S6 | Limiters - English Language  Search modes - Boolean/Phrase | 174 |

## EMBASE

Date searched: 18 May 2018

| # | **Searches** | **Results** |
| --- | --- | --- |
| 1 | exp knee osteoarthritis/ or exp hand osteoarthritis/ or exp hip osteoarthritis/ or exp osteoarthritis/ | 112957 |
| 2 | (((degenerative or noninflammatory or non-inflamatory or non inflamatory) adj2 (Joint or arthritis)) or Osteoarthr*).mp. [mp=title, abstract, heading word, drug trade name, original title, device manufacturer, drug manufacturer, device trade name, keyword, floating subheading word, candidate term word] | 130946 |
| 3 | 1 or 2 | 137620 |
| 4 | exp self care/ | 69593 |
| 5 | exp patient education/ | 103631 |
| 6 | (((self or symptom*) adj2 (car* or help or manag* or directed or monitor* or efficac* or admin*)) or ((health or patient*) adj2 (educat* or information or counsel* or instruct*)) or (management* adj2 (plan or disease*)) or (chang* adj2 (behaviour* or behavior*))).mp. [mp=title, abstract, heading word, drug trade name, original title, device manufacturer, drug manufacturer, device trade name, keyword, floating subheading word, candidate term word] | 811500 |
| 7 | 4 or 5 or 6 | 818003 |
| 8 | exp telemedicine/ | 31058 |
| 9 | exp computer system/ | 24701 |
| 10 | exp educational technology/ | 2823 |
| 11 | exp hypermedia/ | 370 |
| 12 | exp video game/ | 1852 |
| 13 | exp mobile phone/ | 18722 |
| 14 | exp social network/ | 12121 |
| 15 | exp virtual reality exposure therapy/ | 439 |
| 16 | ((Internet or web or computer) adj1 (base* or deliver*)).mp. [mp=title, abstract, heading word, drug trade name, original title, device manufacturer, drug manufacturer, device trade name, keyword, floating subheading word, candidate term word] | 62441 |
| 17 | ((cell* or mobile*) adj3 phone*).mp. [mp=title, abstract, heading word, drug trade name, original title, device manufacturer, drug manufacturer, device trade name, keyword, floating subheading word, candidate term word] | 17106 |
| 18 | (telecare or telerehab* or telemed* or telehealth or teletherap* or e-health or m-health or Ehealth or Mhealth or Computer* or microcomputer* or PC or PCs or Mac or Macs or technolog* or telecommunication or multi-media or multimedia* or hypermedia* or app or apps or Email or E-mail or Mobile or Smartphone or smart-phone or smart telephone or Tablet or cell or hand-held or handheld or VR or virtual reality).mp. [mp=title, abstract, heading word, drug trade name, original title, device manufacturer, drug manufacturer, device trade name, keyword, floating subheading word, candidate term word] | 9632851 |
| 19 | 8 or 9 or 10 or 11 or 12 or 13 or 14 or 15 or 16 or 17 or 18 | 9672850 |
| 20 | exp controlled clinical trial/ | 686016 |
| 21 | crossover procedure/ | 55498 |
| 22 | (Randomized controlled trial or randomized or randomised or randomly or placebo or clinical or Cross over or Crossover or RCT).mp. | 9585880 |
| 23 | 20 or 21 or 22 | 9585880 |
| 24 | animal/ | 1846604 |
| 25 | 23 not 24 | 9458238 |
| 26 | 3 and 7 and 19 and 25 | 950 |
| 27 | limit 26 to english language | 921 |

## CENTRAL

Date searched: 18 May 2018

| **#** | **Search** | **Results** |
| --- | --- | --- |
| 1 | MeSH descriptor: [Osteoarthritis] explode all trees | 4786 |
| 2 | ((degenerative or noninflammatory or non-inflamatory or non inflamatory) near/2 (Joint or arthritis)) or Osteoarthr*:ti,ab,kw (Word variations have been searched) | 10228 |
| 3 | #1 or #2 | 10228 |
| 4 | MeSH descriptor: [Self Care] explode all trees | 5543 |
| 5 | MeSH descriptor: [Education, Nonprofessional] explode all trees | 19715 |
| 6 | MeSH descriptor: [Patient Education as Topic] explode all trees | 8622 |
| 7 | ((self or symptom*) near/2 (car* or help or manag* or directed or monitor* or efficac* or admin*)) or ((health or patient*) adj2 (educat* or information or counsel* or instruct*)) or ((management*) near/2 (plan or disease*)) or ((chang*) near/2 (behaviour* or behavior*)):ti,ab,kw (Word variations have been searched) | 40325 |
| 8 | #4 or #5 or #6 or #7 | 55062 |
| 9 | MeSH descriptor: [Telemedicine] explode all trees | 2241 |
| 10 | MeSH descriptor: [Remote Consultation] explode all trees | 438 |
| 11 | MeSH descriptor: [Teleradiology] explode all trees | 24 |
| 12 | MeSH descriptor: [Telenursing] explode all trees | 27 |
| 13 | MeSH descriptor: [Computer Systems] explode all trees | 5022 |
| 14 | MeSH descriptor: [Educational Technology] explode all trees | 3519 |
| 15 | MeSH descriptor: [Computer-Assisted Instruction] explode all trees | 995 |
| 16 | MeSH descriptor: [Hypermedia] explode all trees | 8 |
| 17 | MeSH descriptor: [Video Games] explode all trees | 533 |
| 18 | MeSH descriptor: [Cell Phone] explode all trees | 1016 |
| 19 | MeSH descriptor: [Virtual Reality Exposure Therapy] explode all trees | 127 |
| 20 | ((Internet or web or computer) near (base* or deliver*)) or telecare or telerehab* or telemed* or telehealth or teletherap* or e-health or m-health or Ehealth or Mhealth or Computer* or microcomputer* or PC or PCs or Mac or Macs or technolog* or telecommunication or multi-media or multimedia* or hypermedia* or app or apps or Email or E-mail or ((cell* or mobile*) near/3 phone*) or Mobile or Smartphone or smart-phone or smart telephone or Tablet or cell or hand-held or handheld or VR or virtual reality:ti,ab,kw (Word variations have been searched) | 183550 |
| 21 | #9 or #10 or #11 or #12 or #13 or #14 or #15 or #16 or #17 or #18 or #19 or #20 | 187165 |
| 22 | MeSH descriptor: [Randomized Controlled Trial] explode all trees | 193 |
| 23 | MeSH descriptor: [Randomized Controlled Trials as Topic] explode all trees | 23029 |
| 24 | MeSH descriptor: [Cross-Over Studies] explode all trees | 33419 |
| 25 | Randomized controlled trial or randomized or randomised or randomly or placebo or clinical or Cross over or Crossover or RCT:ti,ab,kw (Word variations have been searched) | 867678 |
| 26 | 22 or #23 or #24 or #25 | 867719 |
| 27 | MeSH descriptor: [Animals] explode all trees | 8838 |
| 28 | #26 not #27 | 861534 |
| 29 | #3 and #8 and #21 and #28 | 104 |
