# Supplementary material for: Digital Self-Management Interventions for People With Osteoarthritis: Systematic Review With Meta-Analysis
Source: J Med Internet Res. 2020 Jul 20;22(7):e15365. doi: 10.2196/15365 (PMC7428148; doi:10.2196/15365)
Supplement: Multimedia Appendix 2 [file jmir_v22i7e15365_app2.docx]

# Multimedia Appendix 2: Studies Excluded with Reasons

| Reason for exclusion | n. | Study |
| --- | --- | --- |
| Not RCT | 3 | [50-52] |
| Not Self-Management programme | 8 | [53-60] |
| Not digital Intervention | 23 | [61-83] |
| Not OA participants | 4 | [84-87] |
| Study protocol | 2 | [88,89] |
| Conference abstract | 2 | [90,91] |
| Secondary analysis | 1 | [92] |
