# Supplementary material for: Digital Self-Management Interventions for People With Osteoarthritis: Systematic Review With Meta-Analysis
Source: J Med Internet Res. 2020 Jul 20;22(7):e15365. doi: 10.2196/15365 (PMC7428148; doi:10.2196/15365)
Supplement: Multimedia Appendix 3 [file jmir_v22i7e15365_app3.docx]

# Multimedia Appendix 3: BCT Taxonomy Grouping with Explanation and Example texts from studies

| **BCT Taxonomy Grouping/ Study** | **Allen (2010)** | **Allen (2016)** | **Allen (2018)** | **Bossen (2013)** | **Kloek (2018)** | **Skrepnik (2017)** | **Lorig (2008)** | **Rini (2015)** |
| --- | --- | --- | --- | --- | --- | --- | --- | --- |
| **1. Goals and Planning** | 1.1. Goal setting (behavioour) and 1.4 Action planning; *helping participants develop goals and action plans related to osteoarthritis management. …*  *health educator also discussed the participant’s goals and action plans. Participants were asked to identify and write down 1 or more goals related to their osteoarthritis symptoms and management, as well as weekly action plans for achieving these goals. For example, a participant could*  *choose a goal of increasing their walking; corresponding action plans could then specify distances they would attempt to walk on a specific number of days each week.*  1.2 Problem solving;  *The health educator guided participants in overcoming barriers they experienced in* *completing their weekly action plans.* | 1.1. Goal setting (behaviour), 1.5. Review behaviour goal(s); *In addition to the educational content provided, goal*  *setting and / or review (for physical activity and / or*  *weight management) is conducted during each telephone call.*  *… second call is used to review participants’ progress*  *toward goals. All calls use standardized scripts to assist with consistency of information delivery. …*  *counsellors work with participants to determine reasonable short-term goals based on their pain and*  *functional limitations.*  1.2. Problem solving and 1.4. Action planning.  *During each phone call, participants are also guided in*  *the process of selecting specific action plans toward meeting their goal(s). Action plans are written for each of the subsequent weeks between the current and next* *scheduled telephone call.*  *…health goals, and addressing perceived barriers and facilitators of health.* | 1.7. Review outcome goal(s; *At any time during the 8-week period, patients were able to change their exercise routine to one that was more or less difficult. When a more difficult routine was requested, the patient was prompted to complete the mSF-WOMAC.* | 1.1. Goal setting (behavioour); *The BGA program incorporates a baseline test, goal*  *setting, time-contingent PA objectives (ie, on fixed time points),*  *and text messages to promote PA.* | 1.1. Goal setting (behavioour); *…. used for the formulation of a short and*  *long-term goal.* | 1.1. Goal setting (behaviour); *Step Goals*  1.5 Review behaviour goal(s)*; Daily step count vs. Step goal* | 1.4. Action planning. & 1.2. Problem solving; *Action planning; feedback; and methods for solving arthritis related*  *problems (Table 1).* | 1.1. Goal setting (behaviour); *COACHtrack used to set/revise practice goals, Set practice goals, schedule 3 pleasant activities for week.*  1.2 Problem Solving: *Personal Plan for overcoming barriers, Problem-solve with interactive vicarious learning exercise.*  1.4 Action Planning: *Exercise: Create personal plan to use skill that fits personal activities and goals.*  1.5 Review behaviour goal (s);  *Review activity/rest cycling and practices completed in prior week.* |
| **2. Feedback and monitoring** |  | 2.3. Self-monitoring of behaviour; *Patients are asked to review these materials during the intervention period. The booklet also includes worksheets for documenting goals and action plans related to physical activity and weight management, as well as worksheets for documenting practice of cognitive behavioural skills.* | 2.3. & 2.4. Self-monitoring of behaviour and outcome of the behaviour; *patients were asked to record via online logs if they completed the prescribed exercises and if they experienced an increase in knee pain. Progress Tracking, including graphs of pain,*  *function, and exercise over time.* | 2.4. Self-monitoring of outcome(s) of behaviour; *..users are presented with an evaluation form about pain and performance. Pain is assessed*  *with a 10-point Numerical Rating Scale (0 is no pain, 10 is worst possible pain). Performance was measured by three items, …* | 2.3. Self-monitoring of behaviour; *and, at the end*  *of the week, patients were asked to*  *evaluate the execution of their assignments.*  2.6. biofeedback; participant *progress was discussed using online progress Reports (ie, a summary of website visits and patients’ experiences with the exercises).*  *In the last session (week 12), the*  *maintenance of PA was discussed and*  *supported.* | 2.6. Biofeedback *Wearable monitor displays patients daily step count, calories burned and sleep.*  2.2 Feedback on behaviour;  *Daily and monthly cumulative activity trends were available for the patient to review.*  2.4 Self-monitoring of outcome(s) of behaviour; *Patient requested to enter pain and mood data on a one-daily basis.* | 2.2 Feedback on behaviour;  *They also monitor the daily posts of all participants and report inappropriate*  *posts to the investigators.*  2.3. Self-monitoring of behaviour; *Use of logs and medication diaries* | 2.2 Feedback on behaviour; *individualized feedback, e.g.offering suggestions to practice more and tips for doing so when participant responses indicated no or little practice*  2.4. Self-monitoring of outcome(s) of behaviour; *self-monitoring (i.e., a section called COACHtrack used to manage practice reminders, log and view graphs showing progress, and set/revise practice goals),* |
| **3. Social Support** | 3.1. Social support (unspecified); *The health educator guided participants in overcoming barriers they experienced in completing their weekly action plans.* | 3.1. Social support (unspecified); *If participants rate their self-efficacy lower than 7, the counselors recommend they revise this plan so they are more confident they will be able to carry it out.*  3.3. Social support (emotional)  *Motivational interviewing strategies were employed by the counselor throughout the intervention.*  *Cognitive behavioral strategies are also discussed during telephone calls according to the schedule shown in Table 1.*  3.1 Social support (practical) ; *Provider intervention* | 3.2. social support (practical); *If participants did not record in the online logs for a one week period, they*  *received an automated message from the TERC reminding them of the importance of exercising and of recording their exercise information.* | 3.1. Social support (unspecified); *The BGA program incorporates a baseline test, goal*  *setting, time-contingent PA objectives (ie, on fixed time points),*  *and text messages to promote PA.* | 3.1. Social support (unspecified);  *participant* *progress was discussed using online progress Reports (ie, a summary of website visits and patients’ experiences with the exercises).*  *In the last session (week 12), the*  *maintenance of PA was discussed and*  *supported.*  3.2. social support (practical); *The therapist created an e-Exercise account*  *and provided support in the selection of 1 type of PA.*  *Weekly automatic emails informed and*  *reminded patients about new assignments*  *and content ….* |  | 3.2. social support (practical); *Moderators assist participants with the*  *program by reminding them to log on ...* |  |
| **4. Shaping knowledge** | 4.1. Instruction on how to perform the behaviour and 4.2 Information about antecedents; *Providing education related to managing osteoarthritis*  *Symptoms … Participants received written and audio versions of osteoarthritis self-management*  *educational materials, including 10 topics that reflect core aspects of managing osteoarthritis Table 1.* | 4.1. Instruction on how to perform the behaviour; *Participants were given written patient educational materials corresponding to intervention topics. … the first scheduled call is a “content” call (e.g., new educational information is reviewed)* | 4.1. Instruction on how to perform the behaviour and 4.2 Information about antecedents; *The TERC also contained evidence-based educational information to help patients*  *better understand knee OA risk factors, pathogenesis and symptom management.* | 4.1. Instruction on how to perform a behaviour.; *In addition to the weekly modules, information about OA, lifestyle, and videos are provided.* | 4.1. Instruction on how to perform the behaviour and 4.2 Information about antecedents; *Information—each week a new video*  *was generated about OA etiology,*  *pain management, weight management,*  *motivation, medication, and*  *social influences on pain. (motivation)* |  | 4.1. Instruction on how to perform the behaviour; *Interactive, Web-based instruction (The Learning*  *Center);* | 4.1. Instruction on how to perform the behaviour; *Introduce progressive muscle relaxation, ‘mini-practices’ (brief relaxation), activity/rest cycling, pleasant activity scheduling, pleasant imagery and other distraction techniques.* |
| **5 Natural consequences** | 5.1. Information about health consequences; *Participants received written and audio versions of osteoarthritis self-management*  *educational materials,* *including 10 topics that reflect core aspects of managing osteoarthritis Table 1* | 5.1. Information about health consequences; *knowledge of health risks and practices, outcome*  *expectations regarding the costs and benefits of*  *health behaviors,* | 5.1. Information about health consequences; *The TERC also contained evidence-based educational information to help patients*  *better understand knee OA risk factors, pathogenesis and symptom management.* |  |  | 5.1. Information about health consequences; *information on the benefits of walking in a*  *brochure*  5.4 Monitoring of emotional consequences; *Patient enters mood data on a daily basis* |  | 5.4 Monitoring of emotional consequences; *Help user identify positive aspects of experience and address barriers to reinforce use of skill*  5.5 Information about emotional consequences; *Therapeutic rationale (how thoughts, feelings, and actions affect pain through pain “gate”), Describe importance of regular practice* |
| **6. Comparison of behaviour** | 6.1 Demonstration of the behaviour; *Participants also received an exercise video designed for persons with osteoarthritis.* | 6.1 Demonstration of the behaviour; *written patient educational materials corresponding to intervention topics, an exercise video for patients with OA, and an audio CD of relaxation exercises.* | 6.1 Demonstration of the behaviour; *The TERC displayed static images of each individualized*  *exercise routine, as well as motion captured animations of each strength and flexibility exercise to promote correct technique* | 6.1 Demonstration of the behaviour; *In addition to the weekly modules, information about OA, lifestyle, and videos are provided.* | 6.1 Demonstration of the behaviour; *each week the participant was asked to perform*  *2 video-supported exercises on 3 different days…* |  |  | 6.3 Information about others’ approval; *Vicarious learning: Demonstrate how others have changed overdone activities*  6.2. Social comparison; *observation of similar others (characters that represented average members of the target population who modeled use of pain coping skills).*   \|  \| \| --- \| |
| **7. Associations** |  |  |  |  |  | 7.1 prompt/cues;  *Motivational messages* |  |  |
| **8. Repetition and substitution** |  | 8.1 Behavioural practice/ Rehearsal ; *The first call involves education about the*  *specific strategy (including a* *rationale for its application to the management of OA-related pain),*  *basic instruction in the skill, and development of a plan for independent practice and application of the skill.* | 8.7. Graded tasks; *Exercise Progression recommendations, based on serial measures of pain and function" 8. Repetition and substitution; also see above #2.3, #2.4 and #1.7* | 8.7. Graded tasks; *The gradual increase in activities changes the perception that PA is related to pain and reinforces*  *confidence to improve PA performance.* | 8.7. Graded tasks; *Graded activity—the duration of a*  *participant’s chosen PA was gradually*  *increased until the individual*  *short-term goal was met.* |  |  | 8.1 Behavioural practice / rehearsal; *Provide opportunity to practice progressive muscle relaxation, sitting and standing mini-practices practice, pleasant imagery and explore experience.*  8.3 Habit formation; *Describe how to set up practice reminders* |
| **9. Comparison of Outcomes** |  |  |  |  |  |  |  | 9.2 Pros and cons; *Help user identify positive aspects of experience and address barriers to use of skill* |
| **10. Rewards and Threats** |  |  |  |  |  |  |  |  |
| **11. Regulation** |  |  |  |  |  |  | 11.2. Reduce negative emotions; *Managing negative emotions such as anger, fear, and depression…* | 11.2 Reduce negative emotions; *pain coping skills, how thoughts, feelings, and actions affect pain, Introduce concept of negative automatic thoughts and how to identify negative automatic thoughts* |
| **12. Antecedents** |  |  |  |  |  |  | *12.6 Body change; Cognitive symptom management such as relaxation, visualisation, distraction …* | 12.4 Distraction; *Exercise demonstrating how to select and add pleasant activities to routine* |
| **13. Identity** |  |  |  |  |  |  |  |  |
| **14. Scheduled consequences** |  |  |  |  |  |  |  |  |
| **15. Self-belief** |  |  | 15.1. Verbal persuasion about capability; *Performance was measured….. Subsequently, tailored to the answers from the evaluation form, automated text-based messages were generated.* |  |  |  | 15.4. Self-talk; *Cognitive symptom management such as relaxation, visualisation, distraction and self talk* | 15.1. Verbal persuasion about capability; *persuasive arguments regarding participants’ ability to complete tasks* |
| **16. Covert learning** |  | 16.2. Imaginary reward; *knowledge of health risks and practices, outcome*  *expectations regarding the costs and benefits of*  *health behaviors,* |  |  |  |  |  | 16.3. Vicarious learning; *Demonstrate how others have changed overdone activities.* |
