# Supplementary material for: Digital Self-Management Interventions for People With Osteoarthritis: Systematic Review With Meta-Analysis
Source: J Med Internet Res. 2020 Jul 20;22(7):e15365. doi: 10.2196/15365 (PMC7428148; doi:10.2196/15365)
Supplement: Multimedia Appendix 4 [file jmir_v22i7e15365_app4.docx]

Multimedia Appendix 4: Forest plots of Review Comparisons

Contents:

Pain Outcome 1

Physical Function Outcome 2

Disability Outcome 3

Quality of Life Outcome 4

Subgroup Analysis of Exercise Component of the Intervention 4

Sensitivity Analysis 5

## Pain Outcome


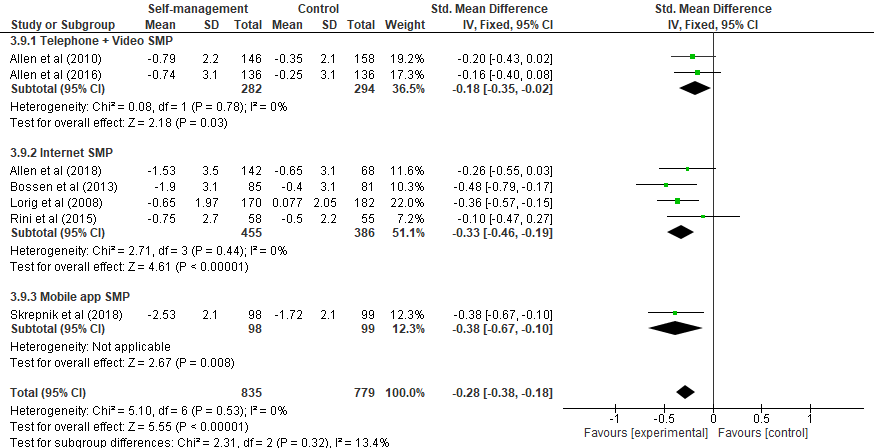


Figure 1: Comparison between digital-based structured self-management intervention and treatment as usual or no intervention control groups in pain reduction at post-intervention time point.


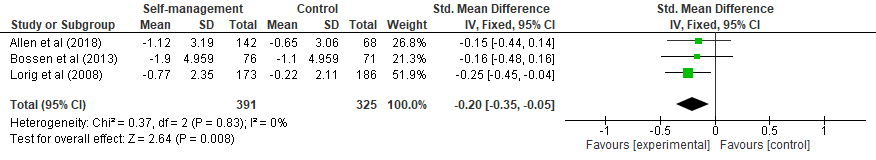


Figure 2: Comparison between digital (internet) self-management intervention and treatment as usual or no intervention control groups in pain reduction at 12-month follow up time point.


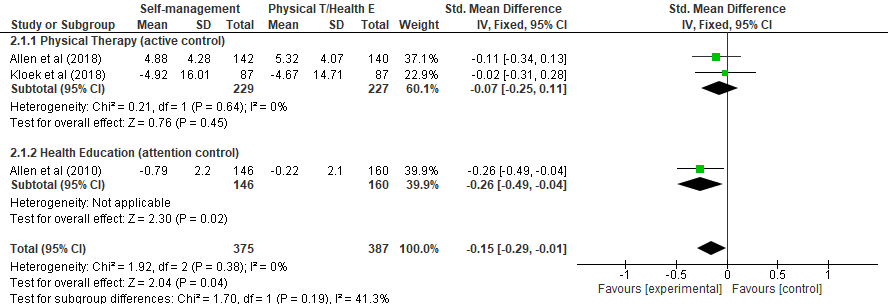


Figure 3: Comparison between digital-based structured self-management intervention against physical therapy or health education treatment groups in pain reduction at post-intervention time point.


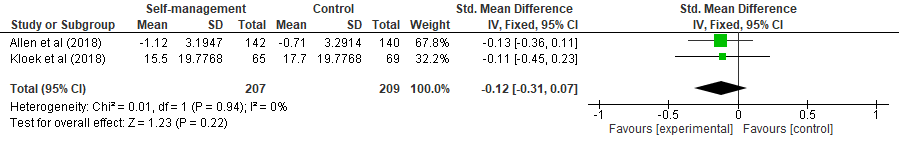


Figure 4: Comparison between digital-based structured self-management intervention and alternative treatment (physical therapy) groups in pain reduction at 12-month follow up time point.

## Physical Function Outcome


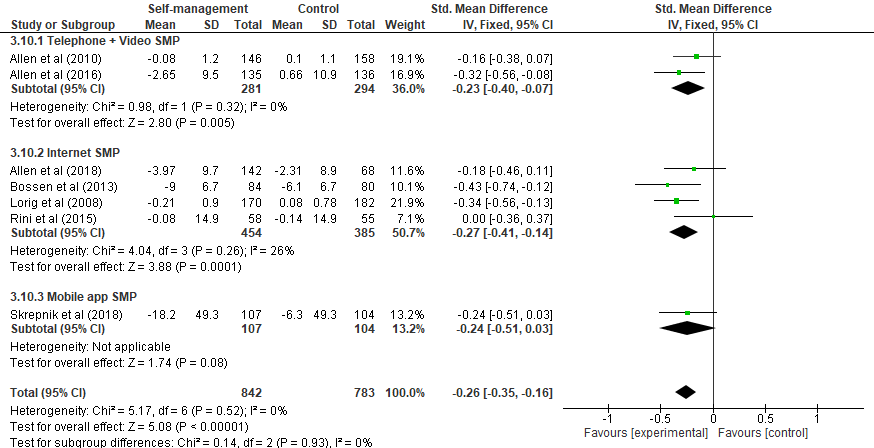


Figure 5: Comparison between digital-based structured self-management intervention and treatment as usual or no intervention control groups in improving physical function at post-intervention time point.


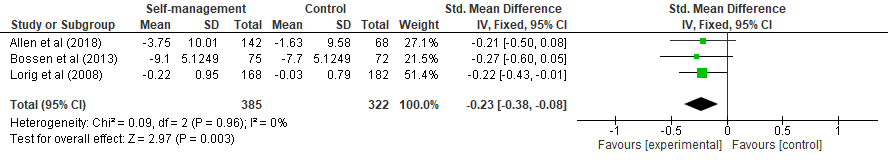


Figure 6: Comparison between digital (internet) self-management intervention and treatment as usual or no intervention control groups in improving physical function at 12-month follow up time point.


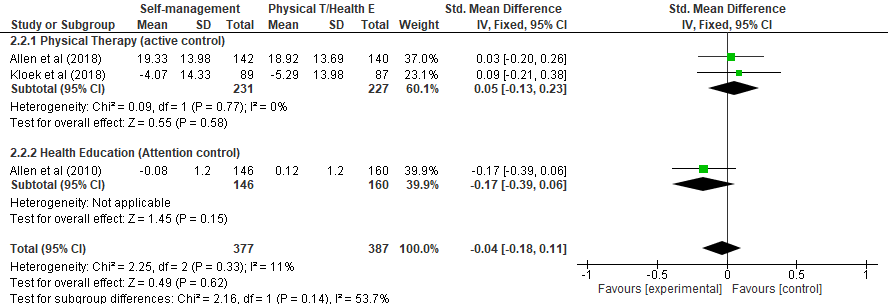


Figure 7: Comparison between digital-based structured self-management intervention against physical therapy or health education treatment groups in improving physical function at post-intervention time point.


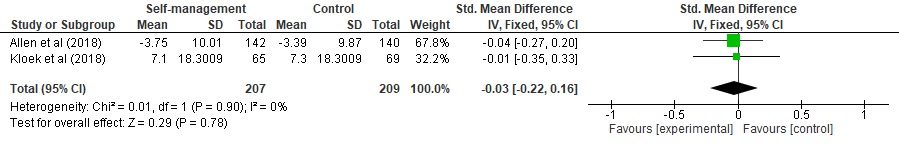


Figure 8: Comparison between digital-based structured self-management intervention and alternative treatment (physical therapy) groups in improving physical function at 12-month follow up time point.

## Disability Outcome


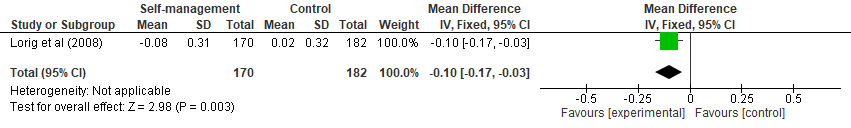


Figure 9: Comparison between web-based self-management program and treatment as usual control groups in disability reduction at post-intervention time point.


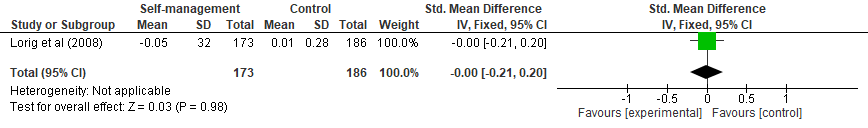


Figure 10: Comparison between web-based self-management program and treatment as usual control groups in disability reduction at 12-month follow up time point.

## Quality of Life Outcome


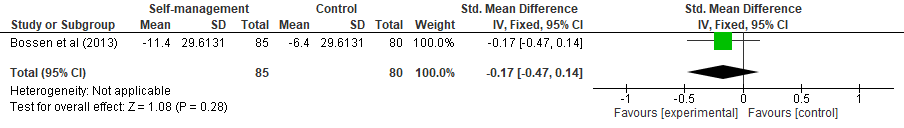


Figure 11: Comparison between web-based self-management program and wait-list control groups in improvement in quality of life at post-intervention time point.


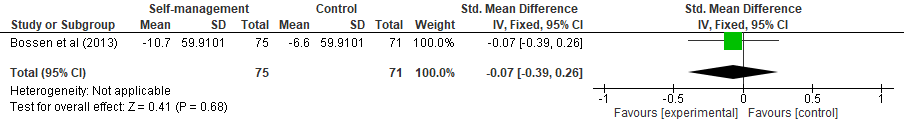


Figure 12: Comparison between web-based self-management program and wait-list control groups in improvement in quality of life at 12-month follow-up time point

## Subgroup Analysis of Exercise Component of the Intervention


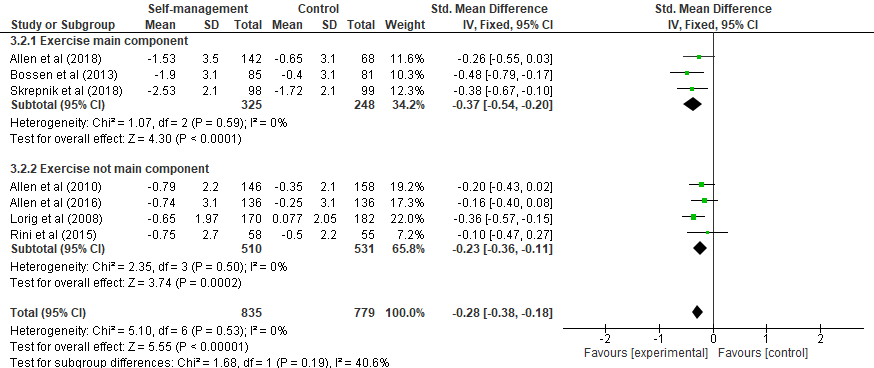


Figure 13: Comparison between studies with exercise as the main component of the intervention with those studies without exercise as the main component of the intervention for pain outcome at post-intervention time point.


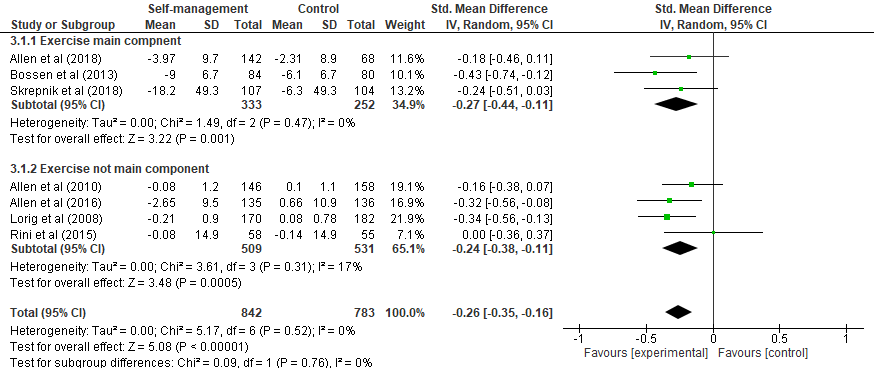


Figure 14: Comparison between studies with exercise as the main component of the intervention with those studies without exercise as the main component of the intervention for physical function outcome at the post-intervention time point.

## Sensitivity Analysis


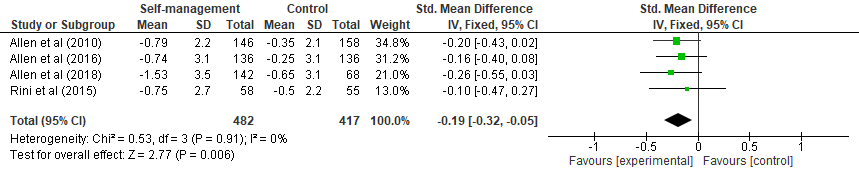


Figure 15: Comparison between digital-based structured self-management intervention and treatment as usual or no intervention control groups in pain reduction at post-intervention time point, in studies with low risk of bias.


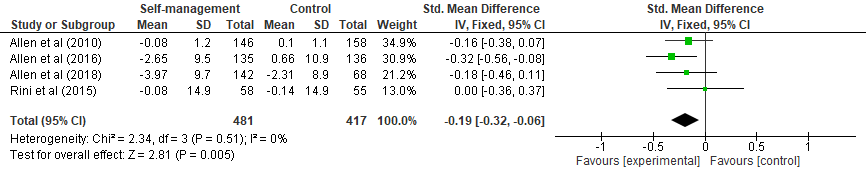


Figure 16: Comparison between digital-based structured self-management intervention and treatment as usual or no intervention control groups in improving physical function at post-intervention time point, in studies with low risk of bias.
